# Improving the Cardiorenal Usability of Endothelin Receptor Antagonism in Albuminuric Chronic Kidney Disease: A Testable Role for SGLT2 Inhibitor–based Mitigation of Fluid Retention

**DOI:** 10.1007/s10557-026-07909-z

**Published:** 2026-07-08

**Authors:** Lucas Maciel de Almeida Corrêa, Luiggi Kevin Virgino Brandão, Yan Roberth Delmiro Silva, Guilherme Diniz Ferreira, Nilson Hitoshi Yoshimoto

**Affiliations:** 1 Faculdade de Medicina de São José do Rio Preto (FAMERP)- Department of Nephrology Avenida Brigadeiro Faria Lima, São José do Rio Preto, Avenida Brigadeiro Faria Lima, Vila São Pedro, São Paulo, 5416, CEP 15090-000 Brazil; 2 Centro Universitário Uninorte (UNINORTE), BR 364, Km 02, Alameda Alemanha, 200, Jardim Europa, Rio Branco, Acre CEP 69915-901 Brazil; 3 Universidade Federal de Alagoas (UFAL), Campus Arapiraca, Avenida Manoel Severino Barbosa, s/n, Bom Sucesso, Arapiraca, CEP 57309-005 Alagoas Brazil; 4 Faculdade de Ciências Médicas de Minas Gerais (CMMG), Alameda Ezequiel Dias, 275, Belo Horizonte, CEP 30130-110 Minas Gerais Brazil; 5 Centro Universitário do ABC (FMABC), Avenida Lauro Gomes, 2000, Vila Sacadura Cabral, CEP 09060-870., Santo André, São Paulo, Brazil

**Keywords:** Chronic kidney disease, Albuminuria, Endothelin receptor antagonists, Sodium-glucose cotransporter 2 inhibitors, Heart failure, Fluid retention, Cardiorenal syndrome

## Abstract

**Purpose:**

Albuminuric chronic kidney disease (CKD) remains a high cardiorenal-risk state despite renin–angiotensin system blockade and sodium–glucose cotransporter 2 inhibitor (SGLT2i) therapy. Endothelin receptor antagonists (ERAs) reduce albuminuria, but clinical use is limited by fluid retention, edema, hemodilution, and concern for heart-failure decompensation. This review examines the testable hypothesis that SGLT2i background therapy may mitigate ERA-associated fluid retention and improve ERA usability in albuminuric CKD without compromising antiproteinuric efficacy.

**Methods:**

We conducted a narrative review prioritizing human studies and practice-relevant data on selective endothelin A receptor antagonism in albuminuric CKD, emphasizing quantitative efficacy signals, fluid retention, heart-failure risk, SGLT2i combination therapy, molecular mechanisms, and therapeutic sequencing and monitoring.

**Results:**

Available evidence supports a biologically coherent but not yet clinically established strategy. SONAR showed that atrasentan reduced kidney events in a responder-enriched population but retained a numerically higher heart-failure hospitalization signal. ZENITH-CKD showed that zibotentan added to dapagliflozin reduced urinary albumin-to-creatinine ratio at 12 weeks, with dose-sensitive fluid-retention events that were more favorable with lower-dose zibotentan. However, evidence remains short-term, agent-specific, and centered mainly on zibotentan plus dapagliflozin. Mechanistically, ERA-associated fluid retention probably reflects interacting tubular, neurohormonal, vascular-permeability, and hemodilution pathways, whereas SGLT2i mitigation may involve natriuresis, plasma-volume contraction, hemoconcentration, tubuloglomerular effects, and non-diuretic cardiovascular mechanisms.

**Conclusion:**

Combined ERA/SGLT2i therapy should be viewed as a mechanistically plausible and clinically testable positioning strategy, not an established treatment paradigm. Broader adoption requires confirmation of durable kidney benefit, cardiovascular safety, real-world feasibility, and reproducible tolerability across eGFR strata and congestion-prone CKD phenotypes.

## Introduction

Renin–angiotensin system (RAS) blockade and sodium–glucose cotransporter 2 inhibitors (SGLT2is) have redefined foundational care in chronic kidney disease (CKD), yet substantial residual risk persists, especially in patients with persistent albuminuria despite optimized therapy [1–5]. Importantly, this residual burden is not purely renal: albuminuric CKD is a cardiorenal risk state in which persistent albuminuria identifies ongoing vulnerability to kidney progression, cardiovascular events, and congestion-prone clinical trajectories [1, 5–7]. In this context, persistent albuminuria should be viewed not merely as an incomplete surrogate response, but as a marker of inadequately controlled pathophysiologic pathways that may justify additional mechanism-based risk-modifying strategies [1, 5, 6].

Accordingly, combination therapy has emerged as a central direction in contemporary cardiorenal medicine, not only because it may enhance antiproteinuric and nephroprotective efficacy, but also because it can improve the therapeutic index of pathway-directed agents through mechanistic complementarity [7, 8]. Not all combinations are equivalent: some merely add effects on intermediate biomarkers, whereas others may also improve tolerability and thereby expand real-world clinical feasibility—a particularly relevant issue when pharmacologic promise is constrained by congestion risk, monitoring burden, or dose-sensitive safety trade-offs [1, 7, 8]. Recent cardiorenal drug literature increasingly emphasizes implementation, therapeutic sequencing, phenotype selection, and measurable cardiovascular–kidney safety outcomes as determinants of therapeutic value in high-risk populations [9–14]. Together, these data support layered residual-risk reduction across the CKD continuum, where clinical usability depends on both efficacy and tolerability [7, 8, 15].

Within this landscape, endothelin receptor antagonists (ERAs) have regained interest in CKD because they consistently reduce albuminuria and, in selected settings, have shown signals of kidney benefit [16–19]. However, broader clinical uptake has remained limited by fluid retention, including edema, weight gain, hemodilution, natriuretic-peptide elevation, and concern regarding cardiac decompensation in a population already vulnerable to volume overload [18, 20–22]. Thus, the central translational question is not whether endothelin blockade is biologically relevant, but whether its kidney benefit can be pursued with acceptable volumetric safety in routine cardiorenal practice, where congestion vulnerability and cardiovascular tolerability help determine therapeutic uptake [21–26].

Accordingly, this review examines a narrower and more practice-relevant question than prior broad overviews of endothelin blockade: whether mitigation of fluid retention may improve the clinical usability and selective positioning of ERA-based therapy in albuminuric CKD without eroding antiproteinuric benefit [7, 22, 23]. We explicitly frame this as a testable therapeutic hypothesis rather than as an emerging clinical paradigm. Rather than re-reviewing the endothelin field in general, we focus on molecular plausibility, quantitative human evidence, limits of extrapolation, practical monitoring, therapeutic sequencing, and implementation challenges required to move from pathway relevance to clinically usable therapy [22–27].

### Scope and Interpretive Approach

This narrative review prioritizes human studies and clinically deployable data on selective endothelin A receptor antagonism in albuminuric CKD, with particular emphasis on evidence informing fluid retention, congestion risk, combination therapy with SGLT2 inhibitors, and practical treatment positioning [18, 21–31]. Rather than providing an exhaustive account of endothelin biology, we intentionally privilege studies with direct relevance to patient selection, dose strategy, safety interpretation, and early monitoring in routine cardiorenal care [21–28].

Because the evidence base remains limited and heterogeneous, this review is not a meta-analysis or practice guideline. It integrates trial-level efficacy and safety data, mechanistic studies, and implementation considerations to clarify what can reasonably be inferred from current evidence, which remains strongest for specific molecules, doses, and trial populations—particularly zibotentan plus dapagliflozin—and should not be generalized to all ERAs, SGLT2 inhibitors, or albuminuric CKD phenotypes [18, 21, 23, 28].

### What this Review Adds to Cardiorenal Drug Therapy

Recent reviews have summarized ERA biology and efficacy signals in CKD [22, 23, 32, 33]. The present review is narrower and more decision-oriented: it treats fluid retention as the principal cardiorenal barrier to ERA usability and asks whether SGLT2is may narrow that barrier enough to inform benefit–risk assessment, patient selection, dose strategy, monitoring, and real-world feasibility [21–28]. This framing aligns with recent cardiorenal drug literature emphasizing implementation, treatment sequencing, heart-failure and kidney outcomes, biomarker interpretation, and congestion-mediated risk as determinants of therapeutic value [9–14].

## The Endothelin Pathway Remains Clinically Relevant in Albuminuric CKD

Endothelin-1 (ET-1) remains clinically relevant in albuminuric CKD because it contributes to intrarenal vasoconstriction, glomerular barrier dysfunction, inflammation, and tubulointerstitial fibrosis [22, 32, 33]. However, in the current therapeutic era, pathway relevance alone is not sufficient to define clinical usefulness [22, 23]. What now matters for clinical cardiorenal practice is whether that biology can be translated into a therapy that remains feasible once volumetric safety, phenotype heterogeneity, and layered background treatment are taken into account [6, 22–26].

The practice-relevant issue is therefore not whether the endothelin pathway matters, but how volumetric safety should shape dose selection, patient suitability, and co-therapy strategies that preserve kidney benefit while narrowing fluid-retention risk [21, 23].

### Molecular Basis of ERA-associated Fluid Retention

ERA-associated fluid retention should not be interpreted as nonspecific edema alone. The kidney endothelin system regulates sodium and water, especially in the collecting duct, where ET-1 signaling interacts with ETA/ETB receptors, nitric oxide, prostaglandins, epithelial sodium transport, vasopressin-sensitive water handling, and medullary tubular function [22, 32–34]. Disruption of this network may alter the balance between antiproteinuric vascular effects and tubular sodium/water homeostasis.

Several mechanisms probably contribute simultaneously. First, selective ETA antagonism may preserve or modify ETB-mediated signaling in ways that affect renal sodium handling and vascular permeability, a complexity that helps explain why fluid retention cannot be reduced to simple “salt retention” alone [34, 35]. Second, changes in collecting-duct sodium transport, including epithelial sodium channel–linked pathways, may promote sodium retention and extracellular volume expansion [34]. Third, vasodilation and neurohormonal counter-regulation may increase renin–angiotensin–aldosterone system and vasopressin activity in susceptible patients, thereby favoring sodium and water retention [22, 34, 35]. Fourth, vascular-permeability effects may contribute to interstitial edema and hemodilution, consistent with clinical signals such as weight gain, lower hemoglobin or hematocrit, edema, and natriuretic-peptide rise [21, 24, 35].

These mechanisms are clinically relevant because they predict a safety phenotype rather than a single adverse event: ERA-associated fluid retention may present as peripheral edema, weight gain, hemodilution, BNP or NT-proBNP elevation, diuretic requirement, or overt heart-failure decompensation depending on baseline cardiac reserve, eGFR, drug exposure, sodium intake, diuretic background, and venous congestion vulnerability [11, 18, 21, 24–26]. This mechanistic heterogeneity supports the need to describe fluid retention quantitatively and operationally, rather than treating it as a generic tolerability limitation.

### Fluid Retention as the Key Cardiorenal Barrier to Wider ERA Use

Fluid retention associated with endothelin receptor antagonism is best viewed as the main cardiorenal barrier to broader use in CKD, because it transforms a biologically attractive antiproteinuric strategy into a therapy whose cardiovascular tolerability may become the rate-limiting step for clinical uptake [18, 20–24, 28]. Although selective endothelin A (ETA) antagonists have shown consistent antiproteinuric effects, their clinical applicability has repeatedly been constrained by edema, weight gain, natriuretic peptide elevations, and hemodilution [16–18, 20, 21, 24]. SONAR made this limitation especially clear by embedding enrichment around early albuminuria response and tolerability, thereby underscoring that the main challenge was not isolated peripheral edema alone, but the broader problem of cardiorenal safety in a population predisposed to volume overload [18, 22, 24].

The quantitative signal from SONAR illustrates this tension. In the enriched responder population, atrasentan reduced the primary kidney composite outcome compared with placebo, but hospitalization for heart failure occurred numerically more often with atrasentan than with placebo, and fluid-retention and anemia-related adverse events were more frequent [18]. Thus, SONAR did not simply demonstrate that endothelin blockade can reduce kidney events; it demonstrated that such benefit becomes clinically interpretable only when response enrichment, exclusion of early intolerability, congestion surveillance, and heart-failure risk are incorporated into the trial design [18, 24].

ZENITH-CKD mechanistic analyses linked extracellular fluid expansion to body weight, BNP, and hemoglobin, supporting structured monitoring rather than a generic adverse-event label [21, 28]. These markers can help determine whether ERA-based therapy remains usable, requires dose adjustment, decongestive intensification, or discontinuation [21, 23, 24].

Fluid-retention risk is not uniform; it varies with drug exposure, kidney function, and baseline clinical context [21, 23]. In ZENITH-CKD, higher zibotentan doses and lower eGFR were associated with greater fluid-retention risk, consistent with higher exposure at lower kidney function [21, 28]. ERA safety in CKD should therefore be viewed as a dose-, eGFR-, exposure-, and phenotype-dependent continuum, particularly in patients with advanced CKD, elevated BNP/NT-proBNP, prior edema, venous congestion, or greater cardiorenal vulnerability [18, 21, 24]. This supports individualized positioning, early monitoring, and explicit consideration of heart-failure vulnerability [18, 21, 23, 24, 28].

### Why SGLT2 Inhibitors may Improve the Benefit–risk Profile and Clinical Usability of ERA-based Cardiorenal Therapy

Within layered cardiorenal risk reduction, the value of an additional agent depends not only on efficacy, but also on whether tolerability preserves clinical implementability [7, 15, 23–26]. In residual albuminuric risk, SGLT2 inhibition is relevant both as foundational therapy and as a potential facilitator of ERA deployment by narrowing the gap between biological efficacy and cardiovascular tolerability [9–14, 23–27, 29].

Beyond nephroprotection, SGLT2is influence natriuresis, sodium balance, extracellular and plasma volume, hemoconcentration, and heart-failure risk [1, 3, 4, 13, 14, 29, 36]. This provides a biologically plausible rationale for attenuating a key ERA safety liability while preserving a guideline-supported cardiorenal foundation [1, 27, 29].

**Fig. 1:**
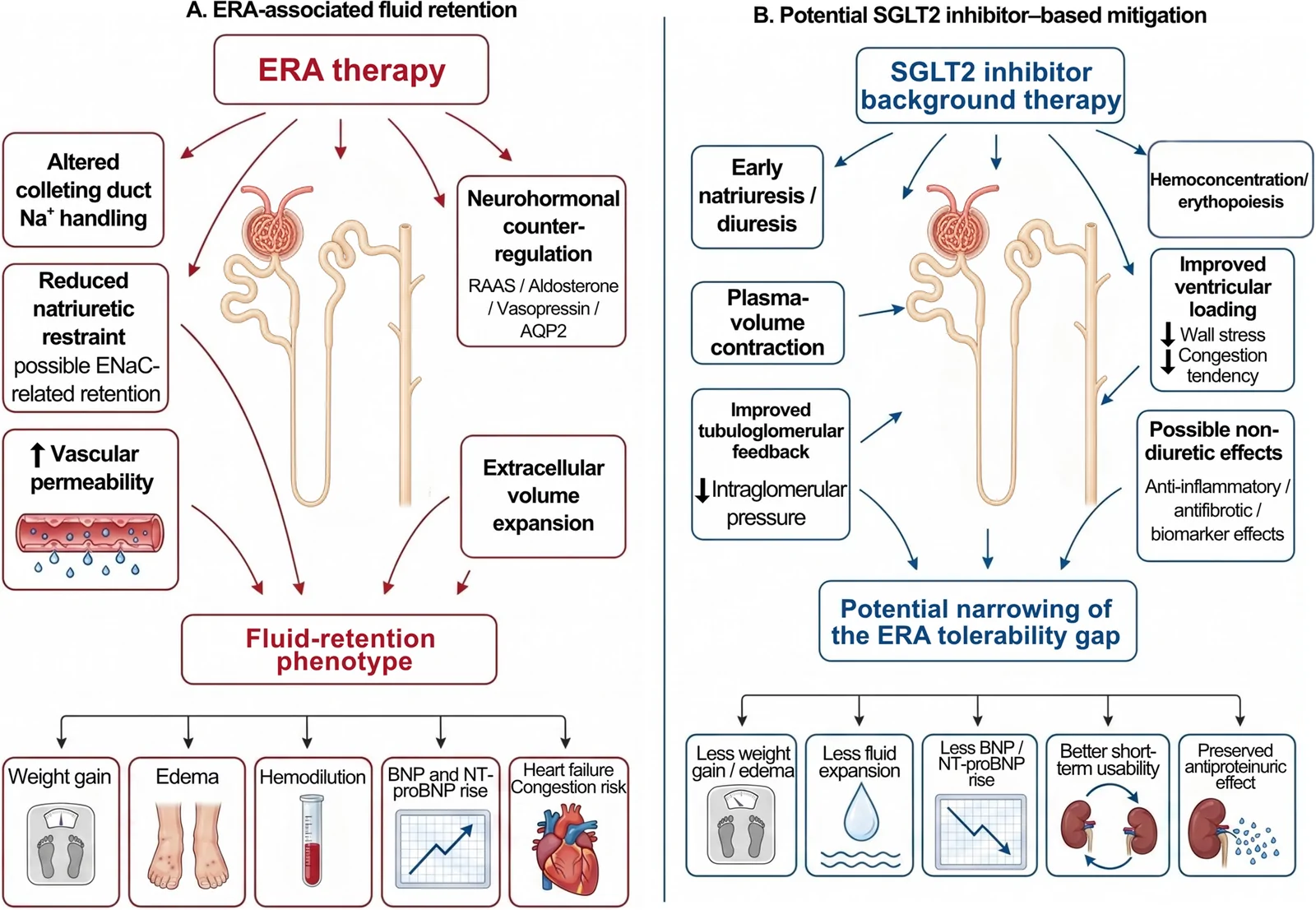
Proposed mechanisms of ERA-associated fluid retention and potential SGLT2 inhibitor–based mitigation. Endothelin receptor antagonist (ERA)-associated fluid retention is presented as a cardiorenal safety phenotype rather than as isolated peripheral edema. During ERA therapy, altered distal tubular and collecting-duct sodium handling, reduced natriuretic restraint, possible ENaC-related sodium retention, neurohormonal counter-regulation involving RAAS/aldosterone and vasopressin/AQP2 pathways, increased vascular permeability, and extracellular volume expansion may converge to produce weight gain, edema, hemodilution, natriuretic-peptide elevation, and heart-failure or congestion vulnerability. SGLT2 inhibitor background therapy may mitigate this liability through early natriuresis and osmotic diuresis, plasma-volume contraction, improved tubuloglomerular feedback with lower intraglomerular pressure, hemoconcentration and erythropoietic effects, improved ventricular loading conditions, and possible non-diuretic cardiorenal effects. This schematic is intended to frame the biological rationale for testing SGLT2 inhibitor–supported ERA regimens in albuminuric CKD; it should not be interpreted as proof that SGLT2 inhibitors abolish ERA-associated fluid retention, that mitigation is purely diuretic, or that these effects apply uniformly across all ERA molecules, doses, or CKD phenotypes. Abbreviations:*AQP2* aquaporin-2, *BNP* B-type natriuretic peptide, *CKD* chronic kidney disease, *ENaC* epithelial sodium channel, *ERA* endothelin receptor antagonist, *NT-proBNP* N-terminal pro-B-type natriuretic peptide, *RAAS* renin–angiotensin–aldosterone system, *SGLT2* sodium–glucose cotransporter 2

SGLT2i-mediated mitigation should not be viewed as purely diuretic. Early natriuresis and osmotic diuresis are relevant, but sustained cardiorenal protection may also involve plasma-volume resetting, hemoconcentration/erythropoiesis, improved tubuloglomerular feedback, lower intraglomerular pressure, improved ventricular loading, reduced wall stress, and anti-inflammatory or antifibrotic effects [14, 29, 36]. Thus, any benefit of SGLT2i/ERA combination therapy may not be attributable solely to volume mitigation (Fig. 1).

The rationale for combining these agents is not limited to additive kidney protection [7, 27]. A more clinically relevant hypothesis is that SGLT2is may improve the therapeutic index of selected ERA regimens by buffering congestion-related risk in a population in whom tolerability often determines whether efficacy can be operationalized [21, 23, 27, 29, 30]. However, this hypothesis should remain anchored to the combinations that have actually been studied rather than generalized across the entire class, especially because fluid retention remains the main translational limitation of selective ETA antagonism [21–23].

Accordingly, the enabling hypothesis should be stated with restraint [23, 27]. SGLT2is may mitigate ERA-associated fluid retention through natriuretic, decongestive, plasma-volume, and possibly myocardial biomarker-related effects, but the most direct human support currently comes from specific combinations, especially zibotentan plus dapagliflozin [21, 27, 28]. Human evidence remains limited; the key question is not whether ERA/SGLT2i combination therapy is established, but whether available studies support a shift from plausibility toward conditional usability in selected phenotypes [21, 28, 31].

### Quantitative Human Evidence for Combined ERA and SGLT2 Inhibition in CKD

A more transparent interpretation requires separating three levels of evidence: first, older ERA trials showing antiproteinuric or kidney-outcome benefit but clinically relevant fluid-retention liability; second, exploratory evidence suggesting that background SGLT2i exposure may attenuate ERA-associated weight gain; and third, prospective short-term evidence from ZENITH-CKD showing that zibotentan added to dapagliflozin reduces albuminuria, with a dose-sensitive fluid-retention signal [18, 21, 28, 30, 31]. Table 1 summarizes the quantitative human evidence supporting this interpretation, separating older ERA efficacy/safety experience, exploratory SGLT2i-mitigation signals, and prospective zibotentan/dapagliflozin data, while highlighting the trial-specific limitations that preclude class-wide extrapolation.

**Table 1:** Quantitative human evidence informing ERA usability and fluid-retention mitigation in albuminuric CKD

Study/program	Population	ERA strategy	Background therapy	Main efficacy signal	Fluid-retention/congestion signal	Key limitation	Practical lesson
ASCEND/avosentan [20]	Overt diabetic nephropathy with high residual proteinuria; on standard care/RAS blockade.	Avosentan 25 or 50 mg/day added to standard treatment.	RAS blockade; pre-SGLT2i era.	Proteinuria reduced by approximately 40%−50% with avosentan versus < 10% with placebo.	Trial stopped early for safety. Adverse-event discontinuation was 19.6% and 18.2% with avosentan versus 11.5% with placebo, dominated by fluid overload and congestive HF; deaths were 4.6% and 3.6% versus 2.6%.	Older higher-exposure ERA strategy; not designed in an SGLT2i-era background; safety penalty limited interpretability.	Antiproteinuric efficacy is not sufficient if congestion toxicity prevents clinical use.
Atrasentan phase 2 diabetic nephropathy [16, 17]	Type 2 diabetes with residual albuminuria despite RAS blockade; eGFR generally 30–75 mL/min/1.73 m² in the 12-week study.	Atrasentan dose-ranging; clinically relevant low-dose strategies included 0.75 and 1.25 mg/day.	Maximally tolerated ACE inhibitor or ARB; pre-SGLT2i era.	UACR decreased by 35% with 0.75 mg/day and 38% with 1.25 mg/day versus placebo; ≥30% UACR reduction occurred in 51% and 55%, respectively.	Weight increased and hemoglobin/hematocrit decreased; peripheral edema was generally manageable, but discontinuation for adverse events was more frequent at 1.25 mg/day.	Short-term biomarker trials; not powered for kidney failure or HF outcomes; no SGLT2i background.	Dose and exposure are central to preserving ERA tolerability.
SONAR/atrasentan [18]	Type 2 diabetes and CKD; enrichment selected patients with albuminuria response and without early fluid-retention intolerance.	Atrasentan 0.75 mg/day versus placebo.	Maximum tolerated RAS blockade; limited SGLT2i use.	Primary kidney outcome occurred in 6.0% with atrasentan versus 7.9% with placebo (HR 0.65; 95% CI, 0.49–0.88).	HF hospitalization was numerically higher with atrasentan: 3.5% versus 2.6% (HR 1.33; 95% CI, 0.85–2.07). Fluid-retention and anemia-related adverse events were more frequent.	Responder/tolerability enrichment improves internal safety but limits generalizability; mostly not an SGLT2i-era trial.	ERA benefit is most interpretable when response, tolerability, and congestion risk are actively selected.
SONAR BNP/HF analysis [24]	Patients exposed to atrasentan during SONAR enrichment and randomized phases.	Atrasentan 0.75 mg/day.	RAS blockade; BNP measured as a safety biomarker.	Not an efficacy trial; analysis focused on HF-risk discrimination rather than albuminuria reduction.	Higher baseline BNP and early BNP rise during atrasentan exposure identified patients at greater subsequent HF risk.	Post hoc risk-stratification analysis; does not define a BNP-guided management algorithm.	BNP or NT-proBNP may help identify congestion-vulnerable patients before overt decompensation.
SONAR SGLT2i post hoc [30]	Small exploratory subgroup with background SGLT2i exposure within SONAR.	Atrasentan with or without background SGLT2i exposure.	RAS blockade; SGLT2i exposure was nonrandomized and uncommon.	Background SGLT2i use was associated with enhanced albuminuria reduction signal.	Background SGLT2i exposure was associated with attenuation of atrasentan-associated weight gain.	Small, exploratory, nonrandomized SGLT2i subgroup; residual confounding likely.	Hypothesis-generating support for SGLT2i-based mitigation, not confirmatory evidence.
ZENITH-CKD/zibotentan + dapagliflozin [28]	Albuminuric CKD with eGFR ≥ 20 mL/min/1.73 m² and UACR 150–5000 mg/g.	Zibotentan 1.5 mg or 0.25 mg plus dapagliflozin versus dapagliflozin plus placebo.	Dapagliflozin 10 mg; ACE inhibitor or ARB at maximum tolerated dose when tolerated.	At 12 weeks, UACR was reduced versus dapagliflozin/placebo by 33.7% with zibotentan 1.5 mg and by 27.0% with zibotentan 0.25 mg.	Fluid-retention events: 18.4% with 1.5 mg + dapagliflozin, 8.8% with 0.25 mg + dapagliflozin, and 7.9% with dapagliflozin/placebo.	Phase 2b, 12-week albuminuria endpoint; not powered for durable kidney outcomes or long-term CV safety.	Low-dose zibotentan on an SGLT2i background provides the clearest current proof of concept.
ZENITH-CKD fluid-dynamics analysis [21]	ZENITH-CKD population with assessment of fluid-retention markers and exposure relationships.	Zibotentan alone and combined with dapagliflozin across dose regimens.	Dapagliflozin or placebo background, depending on trial arm.	Interpreted in the context of preserved short-term albuminuria-lowering signal.	Fluid retention increased with higher zibotentan dose and lower eGFR; dapagliflozin attenuated but did not abolish the signal. Weight, BNP, hemoglobin, extracellular fluid, and exposure were informative markers.	Mechanistic/post hoc analysis; not a clinical outcomes trial; management thresholds remain undefined.	Dose, eGFR, exposure, weight, BNP, hemoglobin, and edema assessment should guide early monitoring.
ZENITH diabetic/non-diabetic analysis [31]	ZENITH-CKD participants with and without type 2 diabetes.	Zibotentan plus dapagliflozin compared with dapagliflozin alone.	Dapagliflozin-based background plus standard CKD care.	UACR reduction with zibotentan/dapagliflozin was directionally consistent in diabetic and non-diabetic CKD subgroups.	Low-dose zibotentan plus dapagliflozin showed a more favorable fluid-retention profile than higher-dose regimens in both clinical contexts.	Subgroup/post hoc nature; short follow-up; no hard outcome confirmation by diabetes status.	Supports biological relevance beyond diabetic CKD but not disease-agnostic implementation.
PRECISION/aprocitentan [39]	Resistant hypertension population; many participants had diabetes and CKD, but not an albuminuric CKD outcome trial.	Dual endothelin receptor antagonism with aprocitentan 12.5 or 25 mg.	Standardized antihypertensive background; not SGLT2i-mitigation design.	Office systolic BP fell versus placebo at 4 weeks; sustained BP effect after withdrawal design.	Edema/fluid retention was dose-dependent: 9.1% with 12.5 mg, 18.4% with 25 mg, and 2.1% with placebo.	Different indication and endpoint; not an ERA/SGLT2i or albuminuric CKD deployability trial.	Fluid retention is a recurring endothelin-pathway safety issue beyond zibotentan and requires active surveillance.

The table separates older ERA efficacy/safety experience, exploratory SGLT2i-mitigation signals, and prospective zibotentan/dapagliflozin evidence. Quantitative values are summarized from the cited studies and should be interpreted as trial-specific rather than class-wide estimates

Abbreviations: *ACE* angiotensin-converting enzyme, *ARB* angiotensin receptor blocker, *BNP* B-type natriuretic peptide, *CKD* chronic kidney disease, *CV* cardiovascular, *eGFR* estimated glomerular filtration rate, *ERA* endothelin receptor antagonist, *HF* heart failure, *NT-proBNP* N-terminal pro-B-type natriuretic peptide, *RAS* renin-angiotensin system, *SGLT2i* sodium-glucose cotransporter 2 inhibitor, *UACR* urinary albumin-to-creatinine ratio

## Early Supportive Signal

An early post hoc analysis from SONAR suggested that background SGLT2i therapy may attenuate atrasentan-associated weight gain and enhance albuminuria reduction [18, 30]. Because this analysis was exploratory, small, and nonrandomized with respect to SGLT2i exposure, it should be interpreted as hypothesis-generating rather than confirmatory [30]. Its main value is not that it proves SGLT2i-mediated protection from ERA-associated fluid retention, but that it generated a clinically testable question later examined prospectively in ZENITH-CKD: whether SGLT2i-based background therapy can preserve antiproteinuric efficacy while narrowing the fluid-retention penalty of ETA antagonism [28, 30].

## What ZENITH-CKD Showed

The ZENITH-CKD trial represents the first pivotal prospective study to investigate the efficacy and safety of combining zibotentan, an ERA, with dapagliflozin, an SGLT2i [28]. This multicenter, double-blind, randomized phase 2b trial enrolled adults with CKD defined by an eGFR of at least 20 mL/min/1.73 m² and a markedly elevated urinary albumin-to-creatinine ratio (UACR) ranging from 150 to 5000 mg/g [28]. Importantly, these pharmacologic interventions were introduced on top of the maximum tolerated dose of angiotensin-converting enzyme inhibitors (ACE inhibitors) or angiotensin receptor blockers (ARBs) [28]. This methodological design reflects contemporary clinical practice and allows a clearer assessment of the true additive benefit of the combination over the established standard of care [1, 28].

ZENITH-CKD showed a clear antiproteinuric signal [28, 31]. At 12 weeks, the addition of zibotentan to dapagliflozin reduced UACR relative to dapagliflozin plus placebo, supporting the view that combined blockade of complementary pathways may enhance short-term albuminuria lowering [28, 31]. Quantitatively, zibotentan 1.5 mg plus dapagliflozin reduced UACR by 33.7% versus dapagliflozin plus placebo, whereas zibotentan 0.25 mg plus dapagliflozin reduced UACR by 27.0% versus dapagliflozin plus placebo at week 12 [28]. At the same time, these data are best interpreted as evidence of biological and clinical signal rather than as proof of durable kidney outcome benefit [23, 28, 31].

Safety remains the critical issue for interpretation [21, 23, 28]. In ZENITH-CKD, fluid-retention events were dose-dependent, with 18.4% of participants in the zibotentan 1.5 mg plus dapagliflozin group, 8.8% in the zibotentan 0.25 mg plus dapagliflozin group, and 7.9% in the dapagliflozin plus placebo group experiencing fluid-retention events [28]. These findings support the concept that careful dose selection within an SGLT2i-based background regimen may improve tolerability, but they do not remove the need for phenotype-based caution, early reassessment, and structured monitoring [21, 23]. The key clinical message is therefore not that dapagliflozin eliminates ERA-associated fluid retention, but that low-dose zibotentan on an SGLT2i background may narrow the safety gap sufficiently to justify further outcome testing [21, 28, 31].

## What the Fluid-retention Analysis Added

ZENITH-CKD fluid-retention analyses showed higher risk with higher zibotentan dose and lower eGFR, consistent with exposure-related volume expansion; dapagliflozin attenuated but did not abolish this signal [21, 28]. These data support the enabling hypothesis but remain mechanistic and short-term, not proof of long-term circulatory safety, hard kidney benefit, or broadly generalizable mitigation [21, 23, 27, 28].

This analysis also reframes ERA therapy as a usability problem rather than an efficacy-only problem: short-term tolerability signals may determine whether a biologically attractive intervention can remain clinically usable in routine cardiorenal care [21, 23–26, 28].

## How Far Can this be Generalized Beyond Zibotentan?

The zibotentan-dapagliflozin experience is best interpreted as proof of principle for a specific combination, not as class-wide validation [21, 23, 28]. Atrasentan and sparsentan support continued relevance of the endothelin pathway across albuminuric kidney diseases, but they do not by themselves establish that SGLT2is uniformly mitigate fluid retention across different ERAs, doses, receptor-selectivity profiles, exposure patterns or CKD phenotypes [18, 19, 37, 38].

The question remains clinically relevant beyond zibotentan because fluid retention is a recurring limitation of ERA-based therapy [18, 20, 22]. Even when an SGLT2i is present, dose-dependent retention signals persist and require monitoring [21, 28]. Evidence from aprocitentan is useful here mainly as a class-safety signal showing that fluid retention remains clinically meaningful across endothelin-directed therapy, rather than as direct support for the specific cardiorenal combination strategy discussed in this review [22, 39].

A balanced interpretation separates pathway relevance from therapeutic generalizability: combined ERA/SGLT2 inhibition should remain molecule-specific, dose-aware, and phenotype-sensitive until broader comparative evidence becomes available [18, 19, 23, 37]. Current evidence does not justify assuming that the tolerability profile of low-dose zibotentan plus dapagliflozin applies to atrasentan, sparsentan, aprocitentan, other ERA doses, or patients not represented in ZENITH-CKD [21, 23, 28, 31, 39].

### The Two Clinically Decisive Questions: ERA after SGLT2i, and ERA when SGLT2i is not Tolerated

Because SGLT2 inhibitors are foundational for most eligible patients with albuminuric CKD, the practical question is whether an ERA provides incremental benefit in patients already receiving RAS blockade and SGLT2i therapy but remaining albuminuric [1, 3, 4, 7, 28]. ZENITH-CKD directly addresses this sequencing scenario by testing zibotentan on a dapagliflozin background [28].

The role of ERA therapy when SGLT2 inhibitors are not tolerated is less certain. Older ERA studies show antiproteinuric and kidney-outcome signals without routine SGLT2i background, but also the fluid-retention liability that limited broader use [16–18, 20, 24]. Such patients require conservative dosing, careful congestion phenotyping, close monitoring, and specialist-level individualized consideration rather than generalizable implementation [18, 21, 23, 24, 28].

### Therapeutic Sequencing in Albuminuric CKD: Where Could ERAs Fit After RAS Blockade, SGLT2 Inhibitors, and MRAs?

A phenotype-sensitive approach becomes clinically meaningful only if it is translated into treatment sequencing. In contemporary albuminuric CKD care, the practical foundation generally includes optimized RAS blockade, SGLT2 inhibition when tolerated, blood-pressure control, dietary sodium reduction, glycemic and lipid risk management, and disease-specific therapy when indicated [1, 3, 4, 7]. In patients with type 2 diabetes and persistent albuminuria, nonsteroidal mineralocorticoid receptor antagonism may also be considered when eGFR and potassium profile permit [8]. Against this background, ERAs should not be positioned as competitors to foundational therapy, but as potential add-on agents for residual albuminuric risk after standard cardiorenal layers have been optimized.

The most plausible future candidate is therefore the patient with persistent albuminuria despite maximally tolerated RAS blockade and SGLT2i therapy, with or without MRA therapy depending on indication and potassium tolerance, who has no overt congestion, no recent heart-failure decompensation, no marked natriuretic-peptide elevation, and an eGFR/exposure profile compatible with the studied regimen [1, 7, 8, 21, 23, 28]. Conversely, patients with unstable heart failure, recent hospitalization for decompensation, clinically evident edema, high BNP or NT-proBNP, advanced volumetric fragility, or very low eGFR should be considered higher-risk candidates in whom the therapeutic window may be substantially narrower [11, 18, 21, 24–26].

The relationship with MRAs is important because SGLT2i/MRA combination therapy is clinically relevant in diabetic CKD and heart failure, and SGLT2 inhibitors may reduce hyperkalemia risk in some contexts [7, 8]. ERA positioning should therefore be part of a broader residual-risk strategy: after RAS blockade and SGLT2i, MRAs address mineralocorticoid-driven inflammation/fibrosis, whereas ERAs target endothelin-mediated albuminuria, vascular dysfunction, and glomerular injury. ERA add-on should depend on whether the expected antiproteinuric gain outweighs patient-specific congestion liability and monitoring burden [7, 8, 21, 23–26, 28].

### From Efficacy Signal to Cardiorenal Therapeutic Positioning

**Fig. 2:**
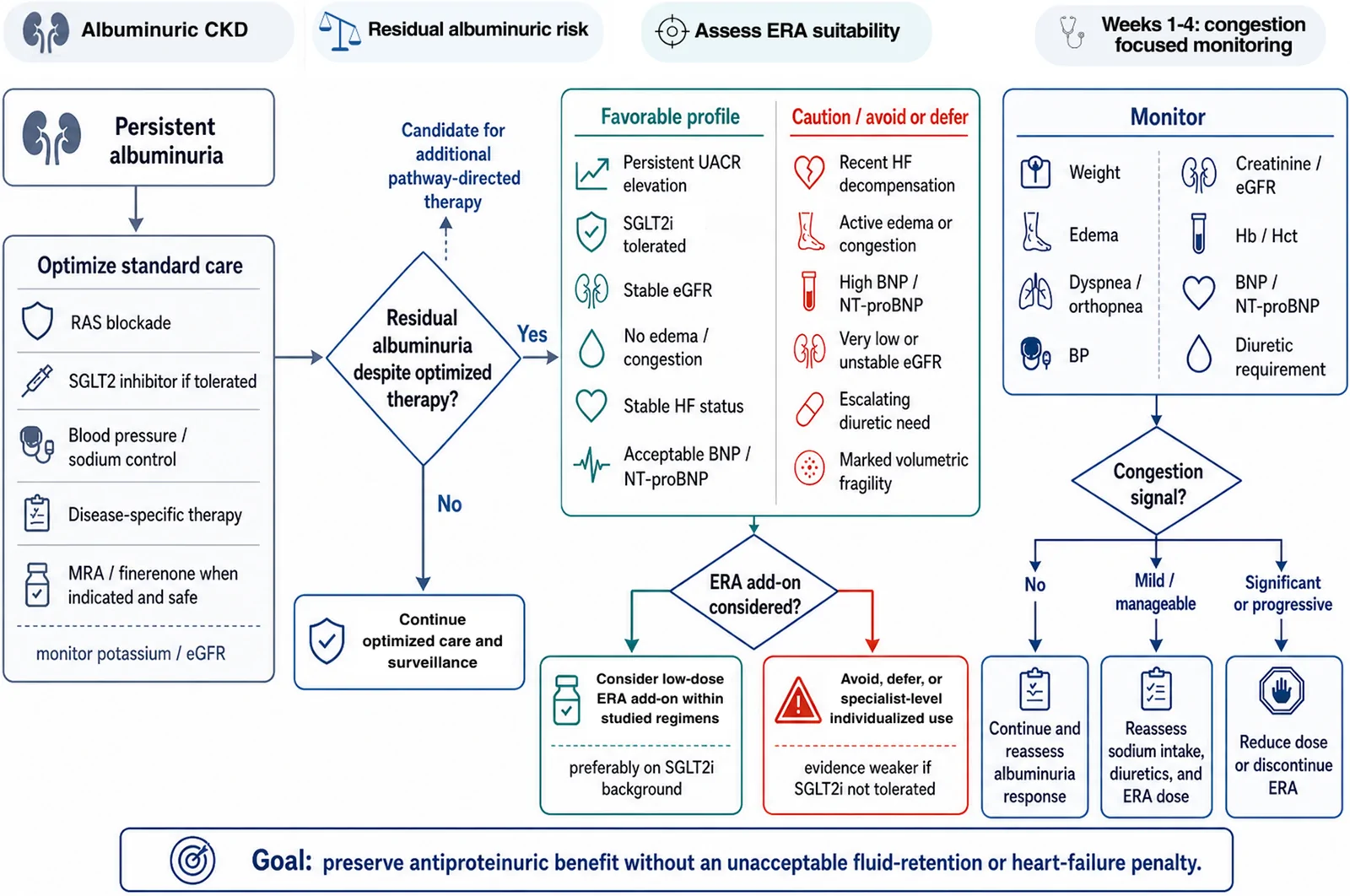
Proposed phenotype-sensitive clinical positioning algorithm for ERA therapy in albuminuric CKD. The algorithm illustrates a practical framework for considering endothelin receptor antagonist (ERA) add-on therapy after optimization of foundational albuminuric CKD care, including renin–angiotensin system blockade, SGLT2 inhibitor therapy when tolerated, blood-pressure and sodium control, disease-specific therapy, and MRA/finerenone when indicated and safe. Persistent albuminuria despite optimized therapy identifies a potential candidate for additional pathway-directed treatment, but ERA suitability should be judged against congestion vulnerability and cardiorenal tolerability. A favorable profile includes persistent UACR elevation, tolerated SGLT2 inhibitor background therapy, stable eGFR, absence of edema or congestion, stable heart-failure status, and acceptable BNP or NT-proBNP. Conversely, recent heart-failure decompensation, active edema or congestion, high natriuretic peptides, very low or unstable eGFR, escalating diuretic requirement, or marked volumetric fragility should prompt avoidance, deferral, or specialist-level individualized consideration. If ERA therapy is considered, available evidence most strongly supports cautious low-dose use within studied regimens, preferably on an SGLT2 inhibitor background; evidence is weaker when SGLT2 inhibitors are not tolerated. Early monitoring during the first 1–4 weeks should focus on weight, edema, dyspnea or orthopnea, blood pressure, creatinine/eGFR, hemoglobin or hematocrit, BNP or NT-proBNP, and diuretic requirement, with treatment reassessment, dose reduction, or discontinuation if congestion signals emerge. This algorithm is intended for clinical positioning and trial interpretation, not as a formal guideline or as evidence of class-wide applicability across all ERAs, doses, or CKD phenotypes. Abbreviations: *BNP* B-type natriuretic peptide, *BP* blood pressure, *CKD* chronic kidney disease, *eGFR* estimated glomerular filtration rate, *ERA* endothelin receptor antagonist, *Hb* hemoglobin, *Hct* hematocrit, *HF* heart failure, *MRA* mineralocorticoid receptor antagonist, *NT-proBNP* N-terminal pro-B-type natriuretic peptide, *RAS* renin–angiotensin system, *SGLT2* sodium–glucose cotransporter 2, *UACR* urinary albumin-to-creatinine ratio

If ERA-based therapy is to become clinically usable, it should be positioned as a cardiorenal strategy integrating residual albuminuric risk, congestion vulnerability, treatment sequencing, monitoring burden, and limits of extrapolation (Fig. 2) [6, 18, 21–24, 28].

## Candidate Patient Phenotype

The most plausible candidate phenotype is the patient with persistent albuminuric CKD despite optimized foundational therapy—ideally including both RAS blockade and an SGLT2i—in whom residual renal and cardiovascular risk remains high enough to justify an additional mechanism-based intervention, but who does not have overt or unstable congestion, recent heart-failure hospitalization or decompensation, marked natriuretic-peptide elevation, or a baseline hemodynamic profile suggesting that even modest fluid retention would carry a disproportionate clinical cost [1, 3, 4, 6, 7, 15, 18, 21, 23–26]. This should be framed as a clinically reasoned positioning rather than a universal indication, because current evidence supports selective deployment in patients with persistent albuminuric risk rather than indiscriminate use across CKD [1, 23, 28].

Concrete clinical scenarios may clarify this point. A favorable future-use phenotype would be a patient with CKD and persistent UACR elevation despite maximally tolerated RAS blockade and SGLT2i therapy, preserved or clinically stable cardiac status, controlled blood pressure, no edema, stable hemoglobin, and low or stable BNP/NT-proBNP. A higher-caution phenotype would be a patient with eGFR near the lower boundary of the studied range, prior edema with vasodilatory therapies, borderline blood pressure, elevated natriuretic peptides, or a history of heart failure without recent decompensation. A generally unfavorable phenotype would include recent heart-failure hospitalization, active edema, escalating loop-diuretic requirement, severe venous congestion, unstable kidney function, or marked natriuretic-peptide elevation [11, 18, 21, 24–26, 28].

Patients warranting greatest caution include those with lower eGFR, clinical congestion or congestion-proneness, prior edema, elevated BNP or NT-proBNP, volumetric fragility, or heart-failure history, because these features may both increase exposure-related fluid-retention risk and lower the threshold for decompensation [18, 21, 23, 24, 28]. Lower kidney function increases zibotentan exposure and fluid-retention risk, and higher baseline or early BNP during atrasentan exposure identifies subsequent heart-failure risk [18, 21, 24]. These patients require conservative dose selection, closer surveillance, and a low threshold for reassessment or de-escalation if congestion signals emerge [21, 23, 24].

### Safety Monitoring and Practical Management of Fluid-retention Risk

Monitoring should be simple, reproducible, and concentrated in the first weeks after initiation or dose escalation, especially in congestion-susceptible patients [21, 23–26, 28]. Serial weight, edema assessment, creatinine/eGFR, hemoglobin/hematocrit, and BNP/NT-proBNP can detect early divergence between antiproteinuric benefit and volumetric tolerance and guide dose adjustment, decongestive intensification, or discontinuation before overt decompensation [21, 23–26, 28].

A practical monitoring approach should include baseline assessment of edema, body weight, blood pressure, eGFR, serum creatinine, hemoglobin/hematocrit, BNP or NT-proBNP, heart-failure history, recent hospitalization, diuretic use, and sodium intake. During the first 2–4 weeks, clinicians should reassess weight gain, edema, dyspnea, orthopnea, blood pressure, creatinine/eGFR, hemoglobin or hematocrit, and natriuretic peptides when clinically appropriate [21, 24, 28]. A rising body weight with edema, falling hemoglobin without bleeding, increasing BNP/NT-proBNP, or escalating diuretic requirement should prompt reassessment of ERA dose, sodium intake, concomitant diuretic strategy, and the need for temporary interruption or discontinuation [21, 23, 24, 28].

Importantly, the evidence base does not yet define an optimal diuretic strategy for ERA-associated fluid retention. No dedicated trial has established whether proactive loop-diuretic adjustment, thiazide-type add-on therapy, mineralocorticoid receptor antagonism, or sodium-restriction protocols reduce heart-failure events or worsening kidney function in ERA-treated CKD patients. Earlier ERA trials and post hoc analyses provide useful safety signals, but they do not answer whether diuretic dose, diuretic class, or decongestive intensity modifies the balance between albuminuria reduction and heart-failure risk [18, 21, 24, 30]. This is a major implementation gap and should be explicitly acknowledged.

A therapy may be mechanistically attractive and still fail in practice if its monitoring burden, congestion liability, or dose sensitivity exceed what routine cardiorenal care can absorb [23–26]. From this perspective, the relevance of SGLT2is is not only biological but operational: if they reproducibly narrow the surveillance burden imposed by fluid retention, they may improve not only tolerability, but also the real-world feasibility of ERA-based therapy [23, 27]. This practical dimension is less emphasized in broader mechanistic reviews, yet it may prove important for actual uptake in cardiorenal care [15, 23–26].

### Implementation Challenges: Regulation, Cost, Access, and Real-world Feasibility

Even if the biological and short-term clinical rationale is compelling, implementation cannot be assumed. First, the most relevant evidence for SGLT2i-enabled ERA therapy is currently centered on short-term zibotentan/dapagliflozin data rather than completed long-term outcome evidence [21, 28, 31]. Therefore, regulatory status and approved indications will determine whether this strategy can be used clinically, and off-label extrapolation across ERAs or CKD phenotypes should be avoided.

Second, cost and access may materially affect feasibility. In layered cardiorenal therapy, cumulative cost, pill burden, monitoring requirements, and payer restrictions may limit uptake even when individual therapies are biologically justified [9, 10]. For ERA-based therapy, early congestion surveillance, natriuretic-peptide testing in selected patients, and clinician familiarity with dose-sensitive fluid retention may further increase implementation burden.

Third, real-world patients may differ substantially from trial populations. Patients with advanced CKD, frailty, multimorbidity, HFpEF, right-sided congestion, pulmonary hypertension, high loop-diuretic requirements, or recent decompensation may be underrepresented in short-term ERA/SGLT2i trials but are precisely the patients in whom fluid retention could become clinically consequential [11, 21, 24–26, 28]. Thus, real-world feasibility should be judged not only by albuminuria reduction, but also by treatment persistence, decongestive medication changes, heart-failure events, outpatient monitoring burden, and discontinuation due to edema or volume expansion.

## What Not to Overstate

This review should not be interpreted as advocating routine ERA/SGLT2i use across CKD [21, 23, 28]. Combined therapy is promising, but the strongest human data remain centered on zibotentan plus dapagliflozin and require confirmation across longer follow-up, broader phenotypes, and real-world settings [21, 23, 28]. Its current value lies in clinical positioning and trial interpretation, not universal implementation [7, 23].

This restrained positioning allows current evidence to inform trial interpretation and phenotype-based reasoning without prematurely converting a conditional strategy into a generalized recommendation [23]. In particular, the current evidence does not establish that SGLT2 inhibitors abolish ERA-associated fluid retention, that the benefits of combination therapy are mediated solely by volume effects, that all ERAs behave similarly, or that patients unable to tolerate SGLT2 inhibitors can be expected to have the same benefit–risk profile as those treated with zibotentan plus dapagliflozin [21, 23, 28, 30, 31].

## Research Agenda: The Questions that Now Matter Most

The next question is what evidence is required before ERA-based therapy can move from conditional usability toward broader cardiorenal incorporation [22–26, 31]. Future studies must define not only efficacy and safety, but also whether phenotype selection, sequencing, and monitoring can support wider implementation [23, 31].

Key gaps include hard kidney benefit, long-term cardiovascular safety, external validity beyond zibotentan/dapagliflozin, early identification of fluid-retention–prone patients, and performance across lower eGFR and heart-failure-prone phenotypes [21, 23, 24, 28, 31]. Trials should also test incremental ERA benefit on RAS/SGLT2i/MRA backgrounds, safety when SGLT2i is not tolerated, and whether BNP/NT-proBNP, hemoglobin, body weight, extracellular volume, or diuretic requirement identify a narrowing therapeutic window [21, 24, 28, 31].

An additional priority is the generation of real-world evidence capable of testing whether the early congestion signals, monitoring strategies, and dose-sensitive safety patterns observed in clinical trials remain reproducible in routine cardiorenal practice, where competing comorbidity burden and variable heart-failure risk may materially alter the benefit–risk balance of therapy [21, 23–26]. Real-world data will be important to test whether ERA-based therapy remains usable amid patient heterogeneity, access limitations, costs, and operational constraints [9–15, 23]. Ongoing studies may help answer these questions, but at present the field remains in a phase of refinement rather than closure [23].

## Disease-specific and Phenotype-specific Caution

The practical appeal of combined ERA/SGLT2 inhibition should not obscure the likelihood that benefit-risk balance will vary across CKD causes and clinical phenotypes [18, 19, 23, 37]. Albuminuric diabetic CKD, IgA nephropathy, and other proteinuric disorders may share pathway-level relevance but still differ in baseline congestion risk, background therapy, regulatory context and acceptable safety trade-offs [18, 19, 37, 38]. For that reason, the present strategy should be interpreted as phenotype-sensitive rather than disease-agnostic, and as informative for clinical positioning rather than as a uniform cross-disease recommendation [23].

This distinction matters because “albuminuric CKD” is not a single therapeutic phenotype. A patient with diabetic CKD, obesity, HFpEF, and elevated natriuretic peptides differs from a younger patient with IgA nephropathy, preserved cardiac reserve, no edema, and persistent albuminuria despite optimized supportive care. Future evidence should therefore report efficacy and safety by CKD cause, eGFR stratum, diabetes status, natriuretic-peptide profile, heart-failure history, and background SGLT2i, MRA, and diuretic use [18, 19, 21, 24, 28, 31, 37, 38].

## Conclusion

Endothelin receptor antagonism remains a credible cardiorenal therapeutic strategy in albuminuric CKD, but fluid retention continues to determine whether that biologic promise can be translated into clinically usable therapy [18, 21–24, 28]. The current relevance of SGLT2is in this setting may lie not only in additive nephroprotection, but also in their potential to improve the benefit–risk profile and clinical implementability of selected ERA regimens by narrowing the gap between biological promise and cardiovascular tolerability [21, 23–28].

At present, combined ERA/SGLT2i therapy should be viewed as a mechanistically coherent and clinically testable strategy rather than an established treatment paradigm. Its strongest human support remains molecule-specific and dose-specific, centered on zibotentan plus dapagliflozin, and its broader use should depend on confirmation of durable kidney benefit, cardiovascular safety, and real-world feasibility across eGFR strata and congestion-prone CKD phenotypes [21, 23–26, 28, 31]. The central message is therefore one of cautious therapeutic positioning: ERAs may become more usable in albuminuric CKD when embedded within SGLT2i-based cardiorenal care, but only if future evidence confirms that antiproteinuric benefit can be achieved without an unacceptable fluid-retention or heart-failure penalty.

## Data Availability

No datasets were generated or analysed during the current study.
